# Compressive Sensing DNA Microarrays

**DOI:** 10.1155/2009/162824

**Published:** 2008-12-22

**Authors:** Wei Dai, Mona A Sheikh, Olgica Milenkovic, Richard G Baraniuk

**Affiliations:** 1 Coordinated Science Laboratory, University of Illinois at Urbana-Champaign, Urbana, IL 61801, USA; 2 Department of Electrical and Computer Engineering, Rice University, Houston, TX 77005, USA

## Abstract

Compressive sensing microarrays (CSMs) are DNA-based sensors that operate using group testing and compressive sensing (CS) principles. In contrast to conventional DNA microarrays, in which each genetic sensor is designed to respond to a single target, in a CSM, each sensor responds to a set of targets. We study the problem of designing CSMs that simultaneously account for both the constraints from CS theory and the biochemistry of probe-target DNA hybridization. An appropriate cross-hybridization model is proposed for CSMs, and several methods are developed for probe design and CS signal recovery based on the new model. Lab experiments suggest that in order to achieve accurate hybridization profiling, consensus probe sequences are required to have sequence homology of at least 80% with all targets to be detected. Furthermore, out-of-equilibrium datasets are usually as accurate as those obtained from equilibrium conditions. Consequently, one can use CSMs in applications in which only short hybridization times are allowed.

## 1. Introduction

Accurate identification of large numbers of genetic sequences in an environment is an important and challenging research problem. DNA microarrays are a frequently applied solution for microbe DNA detection and classification [1]. The array consists of genetic sensors or *spots*, containing a large number of single-stranded DNA sequences termed *probes*. A DNA strand in a test sample, referred to as a *target*, tends to bind or "hybridize" with its complementary probe on a microarray so as to form a stable duplex structure. The DNA samples to be identified are fluorescently tagged before being flushed against the microarray. The excess DNA strands are washed away and only the hybridized DNA strands are left on the array. The fluorescent illumination pattern of the array spots is then used to infer the genetic makeup in the test sample.

### 1.1. Concerns in Classical DNA Microarrays

In traditional microarray designs, each spot has a DNA subsequence that serves as a unique identifier of only *one* organism in the target set. However, there may be other probes in the array with similar base sequences for identifying other organisms. Due to the fact that the spots may have DNA probes with similar base sequences, both specific and nonspecific hybridization events occur; the latter effect leads to errors in the array readout.

Furthermore, the unique sequence design approach severely restricts the number of organisms that can be identified. In typical biosensing applications, an extremely large number of organisms must be identified. For example, there are more than  known harmful microbes, many with significantly more than  strains [2]. A large number of DNA targets require microarrays with a large number of spots. The implementation cost and speed of microarray data processing is directly related to the number of spots, which represents a significant problem for commercial deployment of hand-held microarray-based biosensors.

### 1.2. Compressive Sensing

Compressive sensing (CS) is a recently developed sampling theory for sparse signals [3]. The main result of CS, introduced by Candès and Tao [3] and Donoho [4], is that a length- signal  that is -sparse in some basis can be recovered *exactly* in polynomial time from just  linear measurements of the signal. In this paper, we choose the canonical basis; hence  has  nonzero and  zero entries.

In matrix notation, we measure , where  is the  sparse signal vector we aim to sense,  is an  measurement vector, and the *measurement matrix* is an  matrix. Since , recovery of the signal  from the measurements  is ill posed in general. However, the additional assumption of signal *sparsity* makes recovery possible. In the presence of measurement noise, the model becomes , where  stands for i.i.d. additive white Gaussian noise with zero mean.

The two critical conditions to realize CS are that (i) the vector  to be sensed is sufficiently sparse, and (ii) the rows of  are sufficiently incoherent with the signal sparsity basis. Incoherence is achieved if  satisfies the so-called restricted isometry property (RIP) [3]. For example, random matrices built from Gaussian and Bernoulli distributions satisfy the RIP with high probability.  can also be sparse with only  nonzero entries per row ( can vary from row to row) [5].

Various methods have been developed to recover a sparse  from the measurements  [3,5–7]. When  itself is sparse, belief propagation and related graphical inference algorithms can also be applied for fast signal reconstruction [5].

An important property of CS is its *information scalability*—CS measurements can be used for a wide range of statistical inference tasks besides signal reconstruction, including estimation, detection, and classification.

### 1.3. Compressive Sensing Meets Microarrays

The setting for microbial DNA sensing naturally lends itself to CS, although the number of potential agents that a hostile adversary can use is large, *not all agents* are expected to be present in a significant concentration at a given time and location, or even in an air/water/soil sample to be tested in a laboratory. In traditional microarrays, this results in many inactive probes during sensing. On the other hand, there will always be minute quantities of certain harmful biological agents that may be of interest to us. Therefore, it is important not just to detect the presence of agents in a sample, but also to *estimate* the concentrations with which they are present.

Mathematically, one can represent the DNA concentration of each organism as an element in a vector . Therefore, as per the assumption of only a few agents being present, this vector  is sparse, that is, contains only a few significant entries. This suggests putting thought into the design of a microarray along the lines of the CS measurement process, where each measurement  is a linear combination of the entries in the  vector, and where the sparse vector  can be reconstructed from  via CS decoding methods.

In our proposed microarrays, the readout of each probe represents a probabilistic combination of all the targets in the test sample. The probabilities are representatives of each probe affinity to its targets due to how much the target and probe are likely to hybridize together. We explain our model for probe-target hybridization in Section 2.2. In particular, the cross-hybridization property of a DNA probe with several targets, not just one, is the key for applying CS principles.

**Figure 1:**
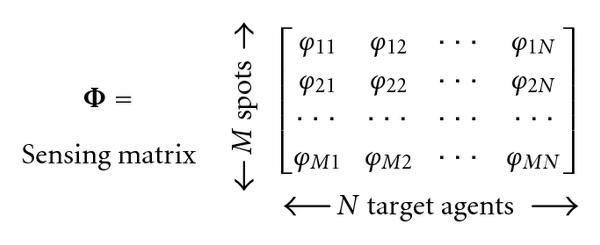
**Structure of the sensing matrix in relation to number of spots and target agents**.

Figure 1 describes the sensing process algebraically. Formally, assume that there is a total number of  possible targets, but that at most  of them are simultaneously present in a significant concentration, with . Let  be the number of measurements required for robust reconstruction according to CS theory. For  and , the probe at spot  hybridizes to target  with probability . The target  occurs in the test DNA sample with concentration . The measured microarray signal intensity vector  equals(1)

Here,  is the sensing matrix, and  denotes a vector of i.i.d. additive white Gaussian noise samples with zero mean.

We note that this probabilistic combination is assumed to be linear for the purposes of microarray design. However, in reality, there is a nonlinear saturation effect when excessive targets are present (see Section 2.4 for details). We take this into account on the reconstruction side, as part of the CS decoding techniques to decipher the combinatorial sensor readout.

Therefore, by using the CS principle, the number of spots in the microarray can be made much smaller than the number of target organisms. With fewer "intelligently chosen" DNA probes, the microarray can also be more easily miniaturized [8–10]. We refer to a microarray designed this way as a CS microarray (CSM).

The CS principle is similar to the concept of group testing [8–11], which also relies on the sparsity observed in the DNA target signals. The chief advantage of a CS-based approach over direct group testing is its information scalability. With a reduced number of measurements, we are able not just to detect, but also to *estimate* the target signal. This is important because often pathogens in the environment are only harmful to us in large concentrations. Furthermore, we are able to use CS recovery methods such as belief propagation that decode  while accounting for experimental noise and measurement nonlinearities due to excessive target molecules [12].

It is also worth to point out the substantial difference between CSMs and the "composite microarrays" designed to reduce measurement variability [13]. In the latter approach, the microarray readouts are linear combinations of input signal components and therefore can be expressed in the form given by (1). However, the  matrix of [13] does typically not satisfy the CS design principles. As a result, the number of required measurements/spots is significantly larger than that of CSMs. On the other hand, the use of the CS principle allows both the robustness of measurements and a significant reduction in the number of spots on the array [14].

### 1.4. Clusters of Orthologous Groups

**Figure 2:**
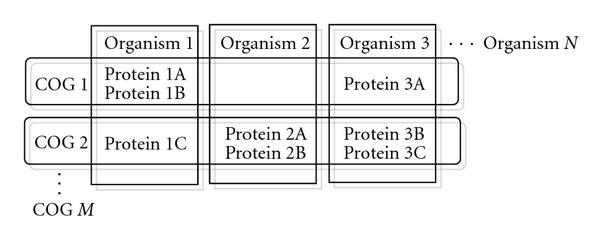
**Block diagram showing a grouping of organisms, their proteins, COGs**.

Note that searching whole genomes of large sets of organisms can be computationally very expensive. As a remedy for classifying the genetic similarity of these organisms, we use the NIH database of clusters of orthologous groups (COGs) of proteins. The COGs database groups the proteins and the corresponding DNA sequences of 66 unicellular organisms into groups ("clusters") based on the similarity of their protein sequences by aligning matching bases in them (see Figure 2 for an illustration). The COGs classification is a phylogenetic classification—meaning that the basis of classification is that organisms of the same ancestral families will demonstrate sequence similarity in their genes that produce proteins for similar function. Since protein sequences can be translated back to the DNA sequences that produced them, a classification of similar proteins is also a classification of DNA similarity.

The COGs database consists of groups of 192, 987 proteins in 66 unicellular organisms classified into 4872 clusters. We use these clusters as a guideline to group targets together. Targets with similar DNA sequences belong to the same group, and can be more easily identified with a single probe. When designing probes, it is important to make sure that the chosen probes align minimally with organisms that do not belong to its group (the "nontargets"). We can use the COGs database with its exhaustive classification to this end, since DNA sequences of an organism whose proteins do not belong to a certain COG will have minimal alignment with DNA sequences of other organisms in that COG. This significantly reduces the computational complexity of the search for good probe sequences.

One limitation in using COGs is that it will constrain design of the  matrix for us. For instance, if we were to choose a set of 10 organisms we are interested in for microarray detection, there are only a finite number of COGs (groups) that these 10 organisms will belong to. We would have to carefully sift through these groups to find the one that best satisfies CS-requirements of , and for each choice, making sure that it is dissimilar enough from the other groups chosen. So on the one hand, using COGs guides our target grouping strategy; on the other hand, it is possible that we might not be able to find enough -suitable COGs to identify all members of the group. Using only a COGs-based approach, we may have to resort to using a  that may not be the best from a CS perspective but simply what nature gives us. Here, however, we only consider an approach using COGs.

A second limitation of COGs is the fact that it is a classification of organisms based on alignments between the *sections* of their DNA that encode for proteins, not entire sequences. Therefore, a point for future exploration would be to work with values from alignments between entire DNA sequences of organisms. Probes selected using such an alignment would be better reflective of the actual probe-target hybridization that takes place in a biosensing device.

However, we are fortunate that prokaryotes such as unicellular bacteria typically have larger percentages of coding DNA to noncoding, and therefore as long as we are interested in the detection of unicellular bacteria, which are prokaryotes, using a COGs-based probe selection is not as much of an issue. On the other hand, eukaryotes have large amounts of noncoding regions in their DNA. This phenomenon is known as the -value enigma [15]: more complex organisms often have more noncoding DNA in their genomes.

### 1.5. CSM Design Consideration

To design a CSM, we start with a given set of  targets and a valid CS matrix . The design goal is to find  DNA probe sequences such that the hybridization affinity between the th probe and the th target can be *approximated* by the value of . For this purpose, we need to go row-by-row in , and for each row find a probe sequence such that the hybridization affinities between the probe and the  targets mimic the entries in this row. For simplicity, we assume that the CS matrix  is binary, that is, its entries have value zero or are equal to some positive constant, say . An entry of positive value refers to the case where the corresponding target and probe DNA strands bind together with a sufficient strength such that the fluorescence from the target strand adhered to the probe is visible during the microarray readout process. A zero-valued entry indicates that no such hybridization affinity exists. How to construct a binary CS matrix  is discussed in many papers, including [16,17], but is beyond the scope of this paper. Henceforth, we assume that we know the  we want to approximate.

The CSM design process is then reduced to answering two questions. Given a probe and target sequence pair, how does one predict the corresponding microarray readout intensity? Given  targets and the desired binding pattern, how does one find a probe DNA sequence such that the binding pattern is satisfied?

The first question is answered by a two-step translation of a probe-target pair to the spot intensity. First, we need a hybridization model that uses features of the probe and target sequences to predict the cross-hybridization affinity between them. Since the CS matrix that we want to approximate is binary, the desired hybridization affinities can be roughly categorized into two levels, "high" and "low," corresponding to one and zero entries in , respectively. The affinities in each category should be roughly uniform, while those belonging to different categories must differ significantly. With these design requirements in mind, we develop a simplified hybridization model in Section 2.2 and verify its accuracy via laboratory experiments, the results of which are presented in Section 2.3. As the second step, we need to translate the hybridization values to microarray spot intensities using a model that includes physical parameters of the experiment, such as background noise. This issue is discussed in Section 2.4.

To answer the second question, we propose a probe design algorithm that uses a "sequence voting mechanism" and a randomization mechanism. The algorithm is presented in Section 3.1. An example of the practical implementation of this algorithm is given in Section 3.2.

## 2. Hybridization Model

### 2.1. Classical Models

The task of accurately modeling the hybridization affinity between a given probe-target sequence pair is extremely challenging. There are many parameters influencing the hybridization affinity. In [18], twelve such sequence parameters are presented, as listed in Table 1.

**Table 1:** 12 parameters used in [18] for predicting hybridization affinities between DNA sequence pairs.

Parameter	Description
	Probe sequence length, Target sequence length
	Probe GC content, target GC content
	Smith-Waterman score: computed from the scoring system used in the SW alignment
	-value: probability that the SW score occurred by chance
	Percent identity: percentage of matched bases in the aligned region after SW alignment
	Length of the SW alignment
	Gibbs free energy for probe DNA folding
	Hamming distance between probe and target
	Length of longest contiguous matched segment in a SW alignment
	GC content in the longest contiguous segment

Many of these parameters () are based on the *Smith-Waterman* (SW) local alignment, computed using dynamic programming techniques [19]. The SW alignment identifies the most similar local region between two nucleotide sequences. It compares segments of all possible lengths, calculates the corresponding sequence similarity according to some scoring system, and outputs the optimal local alignment and the optimal similarity score. For example, if we have two sequences -CCCTGGCT- and -GTAAGGGA-, the SW alignment, which ignores prefix and suffix gaps, outputs the best local alignment 

Another important parameter for assessing hybridization affinity is , the length of contiguous matched base pairs. It has been shown in [18,20] that long contiguous base pairs imply strong affinity between the probe and target. Usually, one requires at least 10 bases in oligo DNA probes for ensuring sufficiently strong hybridization affinity.

Besides the large number of parameters that potentially influence hybridization affinity, there are many theories for which features most influence hybridization and how they affect the process [18,21,22]. A third-order polynomial model using percent identity , as the single parameter, was developed in [21]. More recently, three multivariate models, based on the third-order polynomial regression, regression trees, and artificial neural networks, respectively, were studied in [18].

### 2.2. Our Model for CSM

Different from the above approaches aiming at identifying the exact affinity value, the binary nature of our CS matrix brings possible simplifications. As we have discussed in Section 1.5, we only need to predict whether the affinity between a probe-target pair is either "high" or "low." For this purpose, two set of rules, designed for deciding "high" and "low" affinities, respectively, are developed in this section.

We propose the notion of the best matched substring pair, defined as follows, for our hybridization model.

*Definition 1*. Let  be a DNA sequence. A substring of  is a sequence of the form , where . Consider a given sequence pair  and  and . Let  be a positive integer at most . A pair of substrings of length , one of which is part of  and the other part of , will be denoted by  and , where . 

For a given substring pair of length , the corresponding *substring percent identity* is defined as(2)

where  denotes the Watson-Crick complement of , and  denotes the cardinality of the underlying set.

*The best matched substring pair* of length  is the substring pair with the largest  among all possible substring pairs of length  from the pair of  and .

For a given , *the largest substring percent identity* is the  of the best matched substring pair of length .

For a given  value, the corresponding *best matched length* is defined as(3)

Remark 1. 

For a given , the best matched substring pair is not necessarily unique, while the  value is unique. 

Our definition is motivated by the following observations.

**Figure 3:**
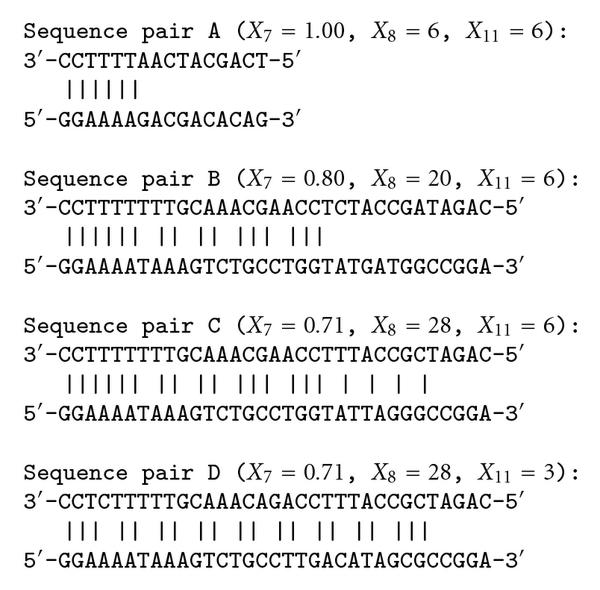
**Aligned sequence pairs from the SW alignment**.

**Figure 4:**
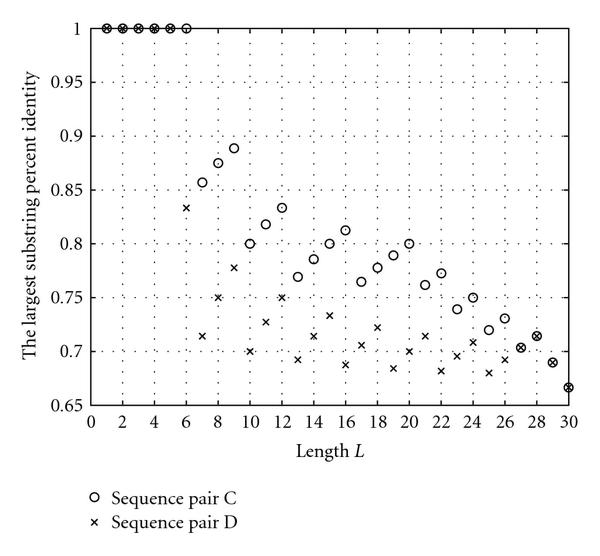
**The s of sequence pairs C and D in Figure 3**.

(1) For hybridization prediction, the parameter percent identity  should be used together with the alignment length . Although the significance of the single-parameter model based on  was demonstrated in [21], we observed that using the  parameter as the sole affinity indicator is sometimes misleading. As an illustration, consider the example in Figure 3. For the sequence pair A, the SW alignment gives  and . For the sequence pair B, the SW alignment gives  and . Though the pair B exhibits a smaller , it obviously has a stronger binding affinity than the pair A, for the aligned part of the pair A is merely a part of the aligned region of the pair B. The same principle holds for the sequence pairs B and C as well. This example shows that besides the percent identity, the *alignment length* is important.

(2) The pair of  and  is not sufficient to predict hybridization affinity. Consider the sequence pairs C and D in Figure 3. Both of them exhibit the same values for the  and  parameters. However, the hybridization affinities of these two pairs are different. To see this, let us refer to Figure 4 which depicts the  values of sequence pairs C and D for different length . It can be observed that for any given , the  value of the sequence pair C is larger than that of the sequence pair D. In other words, the sequences in the former pair match with each other uniformly better than the sequences in the latter pair. The sequence pair C has a larger chance to hybridize than the pair D does. With the same values of parameters  and , the difference in hybridization affinity comes from the distribution of matched bases in the aligned region.

The advantage of using the largest substring percent identities for hybridization prediction is now apparent. The s include all the information contained in the previously discussed , and  parameters; it can be verified that  and that the  is one of the values of s such that . Of course, a list of  provides more detailed information, since it gives both local and global matching information.

Based on the notion of best matched substrings, we propose a set of criteria for CSM probe-target hybridization prediction. A positive-valued entry in the CS matrix suggests that the corresponding probe-target pair satisfies the following two criteria.

(C1) There exists a best matched substring pair of length at least  such that the corresponding substring percent identity satisfies . Alternatively,  such that . Here, both  and  are judiciously chosen parameters.

(C2) Among all the best matched substring pairs with , there should be no pair of length longer than , that is, it should hold that  for all . Again,  has to be chosen properly.

Criterion (C1) guarantees that there is a significantly long substring pair with high-percent identity that ensures strong hybridization affinity. Although criterion (C2) may seem counterintuitive at first glance, it ensures that one single target cannot dominantly hybridize with the consensus probe, that is, the binding affinities between probe-target pairs are roughly uniform.

The probe-target pair associated with a zero entry in the CS matrix satisfies the following two criteria.

(C3) Among all the best matched substring pairs with percent identity at least , there should be no pair of length longer than , that is, .

(C4) Among all the substring pairs matched perfectly (with ), there should be no pair of length greater than , that is,  for all .

Criterion (C3) asserts that there should be no substring pair that has both long length and high-percentage identity. The last criterion, (C4), prevents the existence of a long contiguous matched substring pair which suggests large binding affinity. Again,  and  have to be chosen appropriately.

This model may seem an oversimplification for accurate hybridization affinity prediction. However, in our practical experience with small binary CS matrices (Section 1.5), this model functions properly (see Section 2.3).

The model error can be formulated mathematically as follows. Let us denote the actual affinity matrix by , where the entry  is the affinity between the th probe and the th target,  and . Then the entries of the affinity matrix  are approximation of the entries of the binary CS matrix  of the form(4)

where  is either zero-valued or equal to , and  is the approximation error that is assumed to take small values only. The physical interpretation of  is given in (9). The values of s can be calibrated via lab experiments. Furthermore, the reconstruction algorithm can be designed to be robust to the approximation error.

*Remark 2.* This model can be further refined by introducing weighting factors in the definition of . More precisely, the number of positionally matched base pairs can be replaced by a weighted sum, where C-G and A-T pairs are assigned different values. More accurate model, taking into account nearest-neighbor interaction, can be considered as well [23,24]. These extensions will be considered elsewhere. 

### 2.3. Experimental Calibration of Parameters

Lab experiments were performed to verify our translation criteria (C1)–(C4) and to choose appropriate values for the involved parameters.

The microarray chip employed contains 70 spots distributed within seven rows, each row containing  identical spots for the purpose of providing more accurate readouts. The probe DNA sequences in the first six rows, denoted by probes A, B, , and F, respectively, are 

The last row is a control row, which always gives the maximum fluorescent readout. Here, probes of different lengths are used to test influence of length on hybridization affinity. The target sequences used in our experiments are 

The probe and target sequences were synthesized by *Invitrogen*, with the first three probes purified using the PAG (polyacrylamide gel electrophoresis) method, while all other sequences were purified using the high-performance liquid chromatography method (HPLC). The fluorescent tags of the targets are Alexa 532.

**Figure 5:**
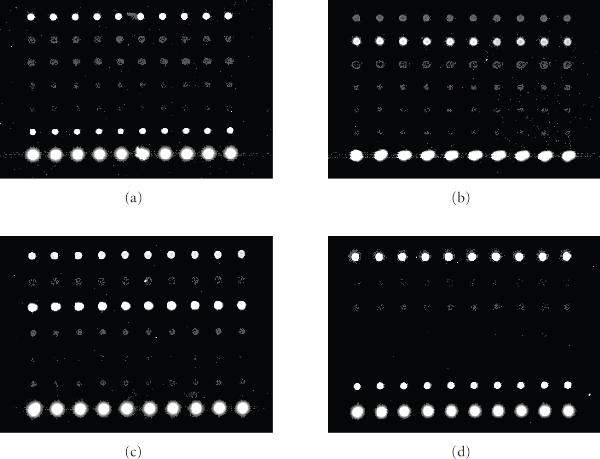
**Microarray readouts**. The readouts (a), (b), and (c) correspond to the targets A, B, and C, respectively, with sixteen-hour incubation, while the readout (d) corresponds to the target A with four-hour incubation.

The experiments proceeded as follows. The first step was to prehybridize our microarray slide. The prehybridization buffer was composed of  TRIS,  Ethanolamin, and  SDS. The printed microarray slide was incubated in the prehybridization buffer at  for 20 minutes. In the hybridization step, we used 1 hybridization buffer (50% formamide, 5X SSC, and 0.1% SDS). We dissolved  target into  hybridization buffer, and then heated the target liquid to  for two minutes to denature. All  target liquid was applied to the prehybridized microarray slide. Then the slide was incubated in a  water bath for 16 hours. In the washing step, we needed three wash buffers: a low-stringency wash buffer containing 1 SSC and  SDS, a high-stringency wash buffer containing 0.1 SSC and  SDS, and a 0.1 SSC wash buffer. After the incubation, we washed the slide (with coverslip removed) with the low-stringency wash buffer (preheated to ), the high-stringency wash buffer, and the SSC wash buffer successively, by submerging the slide into each buffer and agitating for five minutes. Finally, we dried the slide and read it using an Axon 4000B scanner. The same procedure was repeated for each target. The microarray readouts are depicted in Figure 5. A readout associated with target A with shorten incubation time (four hours) is also included (Figure 4(d)).

We study the relationship between these binding patterns and the substring matches. For each probe-target pair, we calculated the corresponding  for each valid  and the s for different  values. Here, we omit most of these results and only list the most important ones in Table 2. We have the following observations.

**Table 2:** Best match substring data.

**** ****	****	****	****	****	****	****
						
						
						

The values in the parenthesis, from the left to the right, are  and . The probe-target pairs corresponding to the bold-font entries exhibit significant microarray readout.

(1) For all sequence pairs exhibiting significant hybridization level, one must have .

(2) For all sequence pairs of which the microarray readout is weak, we have . (For the pair of probe A and Target B, , but the corresponding microarray readout is week.) Consequently,  may be a critical parameter for deciding whether a probe-target pair hybridizes or not.

(3) Among all sequence pairs with weak microarray readouts, the length of the longest contiguous segment is  (the pair of probe C and target A). This fact implies that the probe-target pair may not hybridize even when they have a contiguous matched substring of length .

Based on the above observations, we choose the values of the parameters in the criteria (C1)–(C4) as in Table 3. Here, the values are chosen to allow certain safeguard region. The chosen values are used in our probe-search algorithm (see Sections 3.1 and 3.2). These choices are based on limited experiments, and further experimental calibration/testing is needed to fully verify these parameter choices.

**Table 3:** Chosen values of the parameters in the criteria (C1)–(C4).

Parameter						
Value	0.80	20	25	0.75	16	7

Interestingly, when we reduced the incubation time to four hours such that the full equilibrium has not been achieved, the microarray still gave an accurate readout (see Figure 5(d)). We expect that one can use CSMs in applications for which only short hybridization times are allowed.

### 2.4. Translating Hybridization Affinity into Microarray Spot Intensity

The hybridization affinity values need to be converted into a form that is physically meaningful and reflective of the spot intensities we observe in an experiment. In the case of a one-spot, one-target scenario, the sensing function takes the form(5)

where  is the actual spot intensity we measure for given experimental conditions,  and  are positive hybridization constants,  is the hybridization affinity,  is the target concentration,  presents the mean background noise, and  denotes the measurement noise which is often assumed to be Gaussian distributed with mean zero and variance  [25,26]. This model mimics the well-known Langmuir model, with background noise taken into consideration [26,27].

For the probe-target pairs corresponding to zero entries of  (i.e.,  is close to zero), the measured intensity can be approximated by(6)

Consider the probe-target pairs exhibiting "high" affinities. If the target concentration is small or moderately large, then the microarray readout is approximately(7)

When the target concentration is extremely large, the saturation effect becomes dominant and one has(8)

As a result, in the linear region, the affinity between the th probe and th target is given by(9)

## 3. Search for Appropriate Probes

### 3.1. Probe Design Algorithm

We describe next an iterative algorithm for finding probe sequences satisfying a predefined set of binding patterns, that is, sequences that can serve as CS probes.

The design problem is illustrated by the following example. Suppose that we are dealing with three targets, labeled by , and , and that the binding pattern of the probe and targets is such that the probe is supposed to bind with targets  and , but not with target . Assume next that the hybridization affinities between a candidate probe and targets  and  are too small, while the hybridization affinity between the probe and target  is too large. In order to meet the desired binding pattern, we need to change some nucleotide bases of the probe sequence. For example, consider a particular aligned position of the probe and the targets, the corresponding probe and targets  bases equal to "T," "T," "A," and "A," respectively. In this case, from the perspective of target , the base "T" of the probe should be changed to "A," while from the perspective of target , this "T" base should be changed to any other base not equal to "T." On the other hand, for target  to exhibit strong hybridization affinity with the probe, the identity of the corresponding probe base should be kept intact. As different preferences appear from the perspectives of different targets, it is not clear whether the base under consideration should be changed or not.

We address this problem by using a *sequence voting mechanism*. For each position in the probe sequence, one has four base choices—"A," "T," "C," and "G." Each target is allowed to "cast its vote" for its preferred base choice. The final decision is made based on counting all the votes from all targets. More specifically, we propose a design parameter, termed as *preference value* (PV), to implement our voting mechanism. For a given pair of probe and target sequences, a unique PV is assigned to each base choice at each position of the probe. We design four rules for PV assignment.

(1) If the target "prefers" the current probe base left unchanged, a positive PV is assigned to the corresponding base choice.

(2) From the perspective of the target, if the current probe base should be changed to another *specific* base, then the original base choice is assigned a negative PV while the intended base choice is assigned a positive PV.

(3) If the current base should be changed to *any other* base, then the corresponding base choice is assigned a negative PV while other base choices are assigned a zero PV.

(4) Finally, if a base choice is not included in the above three rules, a zero PV is assigned to it.

The specific magnitude of the nonzero PVs is chosen according to the significance of the potential impact on the hybridization affinity between the considered target and probe. The details of this PV assignment are highly technical and therefore omitted. The interested reader is referred to our software tool [28] for a detailed implementation of the PV computation algorithm.

After PV assignment, we calculate the so-called *Accumulated PV* (APV). For a given base choice at a given position of the probe, the corresponding APV is the sum of all the PVs associated with this choice. The APV is used as an indicator of the influence of a base change in our algorithm; the bases associated with negative APVs are deemed undesirable and therefore should be changed; if the current base of the probe is associated with a positive APV, one would like to leave this base unchanged; if a base choice, different from the current base of the probe, has a positive APV value, one should change the current base to this new choice.

It is worth pointing out the "partly" random nature of the algorithm. In step 5 of our algorithm, whether a current base at a given position is changed or not and which base the current base is changed to are randomly decided. The probabilities with which the current base is changed, and with which a specific base is selected to replace the current base, are related to the magnitudes of the associated APVs. The implementation details behind this randomization mechanism are omitted, but can be found in [28].

This random choice component helps in avoiding "dead traps" that may occur in deterministic algorithms. As an illustrative example, suppose that the intended binding pattern between a probe and all targets except target 1 is satisfied in a given iteration. From the perspective of target 1, the first base of the probe should be changed from "T" to "C." In a deterministic approach, a base replacement must be performed following this preference exactly. However, this base change breaks the desired hybridization pattern between the probe and target 2. In the next iteration, according to the perspective of target 2, the first base of the probe has to be changed back to "T." As a result, this probe base "oscillates" between these two choices of "T" and "C," and the algorithm falls into a "dead trap." In contrast, due to the randomization mechanism in our algorithm, there is a certain probability that the base change does not follow exactly what seems necessary. Dead traps can be prevented from happening or escaped from once they happen.

The algorithm is repeated as many times as the number of probes.

### 3.2. Toy Probe Design Example for 

We describe a proof-of-concept small-scale CSM example. In this example, we have seven target sequences of length 55, listed in Table 4. Also listed are the seven unicellular organisms from which the target sequences are spliced, and the specific genome positions of the targets. Here, we follow the notation convention used by the Kyoto Encyclopedia of Genes and Genomes (KEGG).

**Table 4:** The target nucleotide sequences.

Target 1	
	From Methanothermobacter thermautotrophicus (Mth)—Genome position: complement (142033 142087)
Target 2	
	From Methanococcus jannaschii (Mja)—Genome position: (77481 77535)
Target 3	
	From Methanosarcina acetivorans str.C2A (Mac)—Genome position: (59910 59964)
Target 4	
	From Pyrococcus horikoshii (Pab)—Genome position: complement (1122252 1122306)
Target 5	
	From Archaeoglobus fulgidus (Afu)—Genome Position: complement (365030 365084)
Target 6	
	From Methanopyrus kandleri AV19 (Mka)—Genome Position: complement (1007480 1007534)
Target 7	
	From Thermoplasma volcanium (Tvo)—Genome Position: (636571 636625)

Given the targets, our goal is to design a CSM with three probes that mimics a [3,4,7] Hamming code. The corresponding CS matrix is given as(10)

In the probe-design process, we use the criteria (C1)–(C4) to decide whether a probe-target pair satisfies the corresponding hybridization requirements encoded in the CS matrix (10). The parameters are set according to Table 3. The probe design algorithm (Algorithm 1) for probe selection produced the following outcomes. 

**Figure 6:**
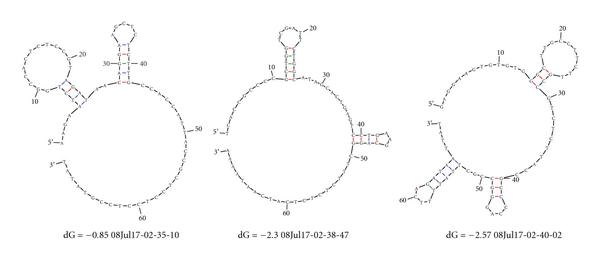
**Secondary structures of the three probes in the toy example**. The predicted structures, from left to right, are corresponding to probes 1, 2, and 3, respectively.

The GC contents for these three probes are 50%, 51.4%, and 51.4%, respectively. The GC contents of the sequences should be of similar value to ensure similar melting temperatures for the duplexes. The secondary structures of these probes can be predicted by using the m-fold package [29] and are depicted in Figure 6. As one can see, all folds have sufficiently long unmatched regions that can hybridize to the targets.

A list of the best matched lengths of the probes and targets is listed in Table 5. According to this table, all probe-target pairs corresponding to entries one of matrix (10) satisfy criteria (C1) and (C2), while all probe-target pairs corresponding to entries zero of matrix (10) satisfy criteria (C3) and (C4). The designed CSM mimics the binary CS matrix (10).

**Table 5:** The best matched lengths of the probes and targets.

	Target 1	Target 2	Target 3	Target 4	Target 5	Target 6	Target 7
Probe 1							
Probe 2							
Probe 3							

The three integers in the parenthesis, from left to right are  and , respectively. The probe-target pairs corresponding to the bold-font entries are designed to have large affinities.

## 4. CSM Signal Recovery

The final step of a CSM process is to estimate the target concentration according to the microarray readout. Recall the signal acquisition model in (5), a signal recovery algorithm specifically designed for CSMs have to take into account the measurement nonlinearity.

Compared to other CS signal recovery methods, *belief propagation* (BP) is the best amenable to incorporate nonlinear measurement. It has been shown that a CS measurement matrix  can be represented as a bipartite graph of signal coefficient nodes s and measurement nodes s [5,12]. When  is sparse enough, BP can be applied, so we are able to approximate the marginal distributions of each of the  coefficients conditioned on the observed data. (Note that the Hamming code matrix  is not sparse. Still, one can use simple "sparsified" techniques to modify  for decoding purpose only [30]). We can then estimate the MLE, MMSE, and MAP estimates of the coefficients from their distributions (we refer to [5,12] for details.)

**Algorithm 1:**Probe design for CSMs.

**Input:** The  target sequences, the row of the intended binding matrix  corresponding to the chosen probe.

**Initialization:** Randomly generate multiple candidates for the probe under consideration. For each candidate, perform the following iterative sequence update procedure.

Iteration:

* * (1) Check the probe's GC content. If GC content is too low, randomly change some "A" or "T" bases to "G" or "C" bases, and vice versa. The GC content afterbase changes must satisfy the GC content requirement.

* *(2) Check whether the probe sequence satisfies the intended binding pattern. If yes, quit the iterations. If not, go to the next step.

* * (3) If an appropriate probe has not been found after a large number of iterations, report a failure, and quit the iterations.

* * (4) For each of the  targets, calculate the PV associatedwith each of the base choice at each position of the probe. Then calculate the APV.

* * (5) Randomly change some bases of the probe sequence so that a potential change associated with a larger APV increment is made more probable.

* * (6) Go back to Step 1.

**Completion:** Check for loop information in the secondary structure of all the surviving probe candidates. Choose the probe with the fewest loops. If more than one such probe exists, randomly choose one of the probes with the shortest loop length.

**Output:** The probe sequence.

**Figure 7:**
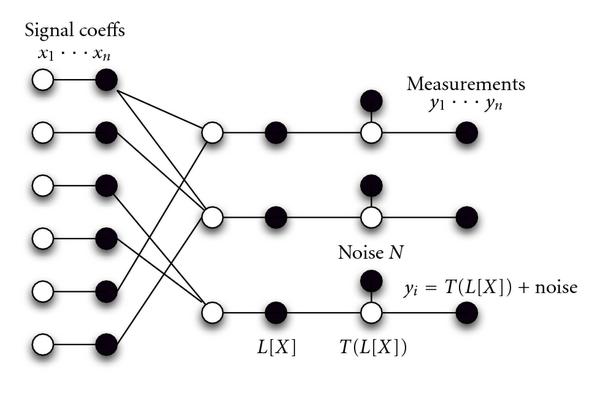
**Factor graph depicting the relationship between the variables involved in CS decoding of the nonlinear intensities**. Variable nodes are black and the constraint nodes are white.

In the context of DNA array decoding, we are given measurement intensities of the spots in the CS microarray, and want to recover the target concentrations s in our test sample. If we abstract the nonlinearity as , and the linear combination of gene concentrations as , we can represent the th spot intensity as(11)

where  is the Gaussian distributed measurement noise. To tailor CS decoding by BP for the nonlinear case, we will account for the nonlinearity  through additional variable nodes, and the measurement noise in the model by noise constraint nodes. The factor graph in Figure 7 represents the relationship between the signal coefficients and measurements in the CS decoding problem for nonlinear measurement intensities  in the presence of measurement noise.

### 4.1. Extracting the Signal from Nonlinear Measurements

Due to saturation effects in the intensity response of the microarray, the nonlinearity acts on  so that recorded measurements will never exceed . We note that due to the presence of measurement noise, the solution is not as simple as inverting the nonlinearity and then applying BP for CS reconstruction.

Our goal is to determine the probability distribution of  at all possible values the true signal values  can take on a grid of sample points, using the measurement intensities  as constraints. The problem then reduces to solving the regular CS signal recovery problem using BP [5]. We note that instead of inverse-mapping  to find , we can calculate the equivalent probabilities of the *transformed* distribution: , by mapping the required sample points for the  distribution to transformed points . At the th measurement node ; the latter probability masses can be picked out at the desired  points. None of the values of  will be evaluated at  values that exceed  by construction. Now, the inverse function is well defined and we can calculate probability masses of  from those of . The problem thus reduces to the regular BP solution for CS reconstruction. This procedure is repeated at each constraint node .

In summary, to "invert" the nonlinearity. 

(1) Transform the sample points  by applying  to get .

(2) For th measurement node , obtain the probability distribution of  which is equivalent to the distribution of . 

(3) Evaluate the probability masses of  at sample grid points .

(4) Calculate probability masses of  from those of  by applying function .

(5) Apply BP for CS decoding as in [5].

### 4.2. Numerical Results

**Figure 8:**
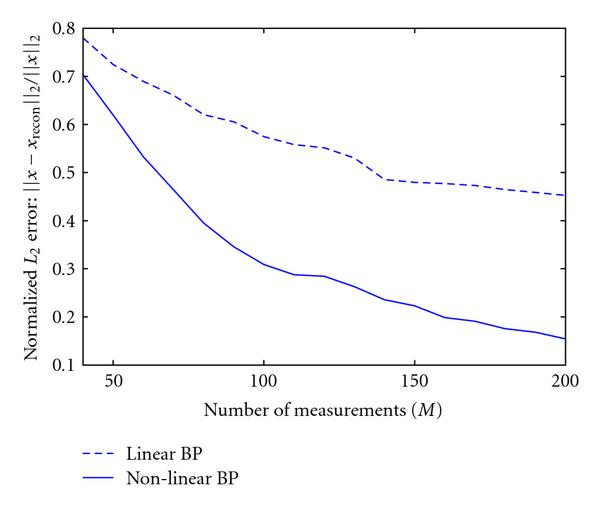
**Plot of normalized  measurement error versus number of measurements for the cases of nonlinear BP-decoding, and BP that ignores the nonlinearity**. Number of signal coefficients  = 200;  =  = 25;

Since the experimental data is currently of relatively small scale, we apply the designed BP algorithm to a set of synthetic data to test the proposed concept. In the computer simulations, we assume that the sparsity of the target concentration signal is 10%. Figure 8 demonstrates the change in  reconstruction error of the signal against the number of measurements (i.e., DNA spots), using our nonlinearly modified BP algorithm, as well as the regular BP decoding algorithm that ignores the nonlinearity. We notice that by taking into account the nonlinearity and reversing it during the decoding process as our modified algorithm does, the  decoding error converges to a smaller value than if we had ignored it. It is important to note that BP appears to be the only CS reconstruction technique that not only meets the requirements of speed in decoding, but can also incorporate the nonlinearity in the measurement prior with ease.

## 5. Conclusion

We study how to design a microarray suitable for compressive sensing. A hybridization model is proposed to predict whether given CS probes mimic the behavior of a binary CS matrix, and algorithms are designed, respectively, to find probe sequences satisfying the binding requirements, and to compute the target concentration from measurement intensities. Lab experimental calibration of the model and a small-scale CSM design result are presented.
